# Antioxidant and inflammatory potential of diet among women at risk of cervical cancer: findings from a cross-sectional study in Italy

**DOI:** 10.1017/S1368980021001944

**Published:** 2022-06

**Authors:** Andrea Maugeri, Martina Barchitta, Roberta Magnano San Lio, Aurora Scalisi, Antonella Agodi

**Affiliations:** 1 Department of Medical and Surgical Sciences and Advanced Technologies “GF Ingrassia”, University of Catania, Via Santa Sofia 87, 95123 Catania, Italy; 2 Cervical Cancer Screening Unit, Azienda Sanitaria Provinciale of Catania, Catania, Italy; 3 The Italian League against Tumors (LILT), Florence, Italy

**Keywords:** Diet, Nutritional epidemiology, Oxidative stress, Inflammation, HPV infection

## Abstract

**Objective:**

To evaluate the association of Composite Dietary Antioxidant Index (CDAI) and Dietary Inflammatory Index (DII) with the prevalence of high-grade cervical intraepithelial neoplasia (CIN).

**Design:**

A cross-sectional study was conducted on women with abnormal Papanicolaou test, who underwent high-risk human papillomavirus (HPV) screening and histological test through colposcopy. Dietary data were collected using a FFQ and used to assess both CDAI and DII.

**Setting:**

Women were recruited from 2012 to 2015 at the Cervical Cancer Screening Unit of the ‘Azienda Sanitaria Provinciale’ of Catania (Italy).

**Participants:**

The study included 539 women with a mean age of 40·2 years, who were classified as cases (*n* 127 with CIN2 or more severe lesions) and controls (*n* 412 with normal cervical epithelium or CIN1).

**Results:**

Although we observed a lower proportion of HPV-positive women among those with higher CDAI (*P* < 0·001), the index was not associated with the diagnosis of CIN2 or more severe lesions. By contrast, women with medium or high DII showed higher odds to be diagnosed with CIN2 or more severe lesions than those with low DII (OR = 2·15; 95 % CI 1·11, 4·17; *P* = 0·024 and OR = 3·14; 95 % CI 1·50, 6·56; *P* = 0·002, respectively), after adjusting for age, HPV status, educational level, BMI, smoking status, parity, use of oral contraceptives and supplements.

**Conclusions:**

Our findings suggested that a pro-inflammatory diet might be associated with an increased risk of CIN2 and more severe lesions. However, further prospective studies should be encouraged to support this evidence.

Cervical cancer (CC) is a major concern for public health, representing the fourth most common cancer in women after breast, colorectal and lung cancers^(1)^. Although the persistency of high-risk human papillomavirus (HPV) infection represents a necessary event for tumour transformation^(2)^, it alone is not sufficient for the progression of cervical intraepithelial neoplasia (CIN) to CC^(3–7)^. For this reason, further physiological and pathological mechanisms might be involved in the aetiology of CC, such as chronic inflammation and oxidative stress. Interestingly, the interaction between chronic inflammation and oxidative stress might trigger HPV-associated carcinogenesis and sustain tumour progression^(8–10)^. In fact, a chronic inflammatory state leads to the production of reactive oxygen species and the release of pro-inflammatory factors (e.g. IL-1 and IL-6, tumour necrosis factor-*α* and interferon *γ*)^(3)^. Moreover, chronic inflammation in HPV-infected cells seems also associated with a decreased level of antioxidants^(11)^. This significantly reduces the antioxidant activity against reactive oxygen species at cellular level, and hence increases oxidative DNA damage^(8,9)^.

The interaction between chronic inflammation and oxidative stress can also provide a partial explanation of biological aspects underpinning the risky effect of several factors involved in CC development (e.g. cigarette smoking, dietary deficiencies, sedentary lifestyle and use of contraceptives)^(4,8,9,12–14)^. Among these, dietary factors have been estimated to contribute to 20–60 % of cancers^(15)^. Accordingly, it is now well established that consuming healthy foods – such as fruit, vegetables, whole grains, and fish – protects against several non-communicable diseases, such as cardiometabolic diseases and certain types of cancer. This evidence has been proven by different studies evaluating the intake of single foods, specific dietary indexes and the adherence to dietary patterns derived a priori or a posteriori^(12,16–20)^.

In this scenario, several studies have been conducted to investigate the effect of foods, nutrients and dietary patterns against HPV infection and CC risk^(3,7,8,12,21–33)^. Among them, there were some epidemiological studies, systematic reviews and meta-analyses focusing on dietary factors with antioxidant anti-inflammatory properties^(8,21–26,30,31,33)^. Despite increasing evidence in this field of research, the combined effect of foods and nutrients with antioxidant and anti-inflammatory activity remains to be clarified. To our knowledge, only the study by Sreeja and colleagues suggested that adherence to a pro-inflammatory diets increased the risk of CC, warranting further studies to confirm their findings^(26)^. With respect to dietary antioxidant intake, instead, there is a lack of supporting evidence. In a previous study on women with normal cervical epithelium, we demonstrated a negative association between dietary antioxidant intake and the risk of HPV infection^(25)^. Here, we aim to evaluate the associations of the Composite Dietary Antioxidant Index (CDAI) and the Dietary Inflammatory Index (DII) – two of the most used indexes to assess antioxidant and inflammatory potential of diet – with the overall prevalence of CIN2, CIN3 and carcinoma *in situ* among Italian women.

## Methods

### Study design

The present cross-sectional study was conducted on all women who consecutively underwent CC screening at the Cervical Cancer Screening Unit of the Azienda Sanitaria Provinciale of Catania (Italy) from 2012 to 2015. The following inclusion criteria had to be satisfied: (1) women with abnormal Papanicolaou test; (2) who were subjected to high-risk HPV screening and (3) histological test through colposcopy. At recruitment, the screening for high-risk HPV was performed using the Digene HC2 HPV DNA Test (Qiagen, Milan, Italy) and allowed to classify women in HPV-negative and HPV-positive. The Digene HC2 HPV DNA Test was applied to cervical specimens and used the signal-amplified nucleic acid hybridisation for the qualitative detection of the following 13 types of high-risk HPV: 16, 18, 31, 33, 35, 39, 45, 51, 52, 56, 58, 59 and 68. At the same time, women were also classified according to histological diagnosis into those with normal cervical epithelium or CIN1 (from now on referred to as controls) and those with a diagnosis of CIN2, CIN3 or carcinoma *in situ* (from now on referred to as cases). A full description of study protocols and characteristics of the study population has been previously reported^(12,25,34)^.

### Data collection

At recruitment, women were interviewed by trained epidemiologists to collect information on sociodemographic characteristics, behaviours and anthropometric measures. Face-to-face interviews were conducted with the aid of a structured questionnaire. With respect to sociodemographic characteristics, women were classified as having a low (primary education) or medium-high (secondary and tertiary education) educational level, and as being employed or unemployed (i.e. including students and housewives). Regarding behaviours, women were classified as current or non-current smokers (i.e. including both former and never smokers), as well as in users or non-users of oral contraceptives and supplements (i.e. including multivitamin and/or multimineral supplements). Women were also asked to report their height and weight that were used to calculate BMI according to the WHO criteria^(35)^.

### Dietary assessment

At recruitment, we also collected dietary data using a semi-quantitative FFQ, as fully described elsewhere^(12,36–39)^. In brief, the FFQ consisted of 95 items (i.e. both foods and beverages) for which frequency of consumption and portions size were collected. The daily dietary intake was obtained by multiplying the daily frequency of consumption by the portion size for each item. Here, the FFQ was used to calculate daily intake of foods and nutrients with antioxidant and anti- or pro-inflammatory properties during the month before the recruitment. Specifically, nutrient composition of each food was determined using the US Department of Agriculture (USDA) Food Composition Database.

Prior to further analyses, women with extreme total energy intake (i.e. those in the 5th or 95th percentile) were excluded. Dietary intakes of remaining women were further adjusted for total energy intake using the residual method^(40)^. Based on energy-adjusted nutrient intakes, we computed two dietary indexes which reflected the cumulative dietary antioxidant intake and the inflammatory potential of diet. Specifically, we adapted the CDAI proposed by Wright and colleagues^(41)^ and the DII developed by Shivappa and colleagues^(42)^. The CDAI was calculated as the sum of *Z*-scores of the dietary intakes of Zn, Se, Mg, vitamin A, vitamin C, vitamin E, *β*-carotene and flavonoids. With respect to the DII, we first calculated the *Z*-score for each dietary factor. Next, *Z*-scores were converted into percentile scores, with a symmetrical distribution (i.e. mean 0 and range from −1 to 1). The DII was then calculated as the sum of the products of percentile scores of dietary factors and their respective inflammatory effect scores. Inflammatory effect scores were previously derived from a systematic review of studies evaluating the inflammatory potential of forty-five dietary factors based on six inflammatory markers (i.e. C-reactive protein, IL-1*β*, IL-4, IL-6, IL-10 and tumour necrosis factor-*α*)^(42)^. In the current study, we used the following thirty-three of the forty-five dietary factors considered in the original DII: alcohol, vitamin B_12_, vitamin B_6_, *β*-carotene, coffee, carbohydrates, cholesterol, energies, total fats, fibre, folic acid, Fe, Mg, MUFA, niacin, proteins, PUFA, riboflavin, saturated fats, Se, thiamin, trans fats, vitamin A, vitamin C, vitamin D, vitamin E, Zn, black/green tea, flavan-3-ol, flavones, flavonols, flavonones and anthocyanidins. Finally, women were classified according to the tertile distribution of each dietary index, so that the cumulative dietary antioxidant intake and the pro-inflammatory potential increased from the 1st to the 3rd tertile.

### Statistical analysis

All statistical analyses were performed on Stata (version 16.0). Prior to analysis, the Kolmogorov–Smirnov test was applied to test the normality of quantitative variables. Descriptive statistics were used to characterise the study population, in terms of percentages for qualitative variables and median with interquartile range for quantitative variables. Comparisons were performed using Mann–Whitney *U* test or the Kruskal–Wallis test for quantitative variables, and the chi-square test for qualitative variables. The Bonferroni correction was applied to adjust for multiple comparisons. Logistic regression models were applied to evaluate the association of dietary indexes with histological diagnosis of CIN2 or more severe lesions. For the DII index, results were reported as OR and 95 % CI of women in the 2nd or 3rd tertile compared with those in the 1st tertile. For the CDAI index, the reference group was constituted by women in the 3rd tertile. Model 1 was adjusted for age and HPV status, while Model 2 was further adjusted for educational level, BMI, smoking status, parity, use of oral contraceptives and supplements. We also tested for interaction of DII or CDAI indexes with the above-mentioned covariates. All statistical tests were two-sided with a significance level of 0·05.

## Results

### Study population

**Fig. 1:**
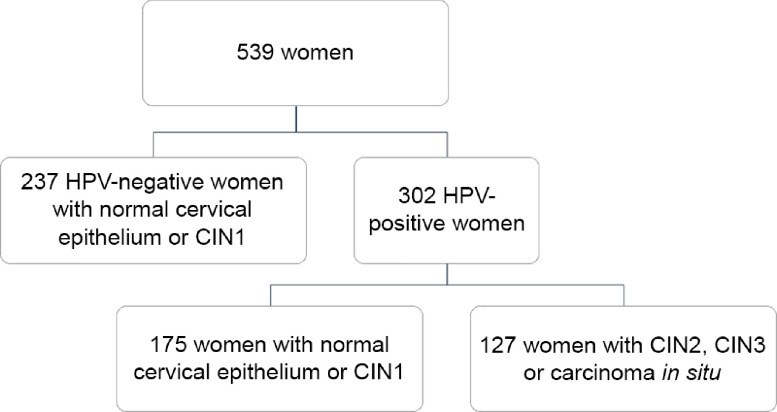
Description of the study population according to high-risk HPV status and histological diagnosis. CIN, cervical intraepithelial neoplasia; HPV, human papillomavirus

Overall, the current analysis included 539 women (mean age = 40·2 years; sd = 10·0 years), who satisfied the inclusion criteria. Figure 1 shows the composition of the study population according to high-risk HPV status and histological diagnosis. Specifically, 44·0 % of women (*n* 237) were HPV-negative and none of them was diagnosed with CIN2, CIN3 or carcinoma *in situ*. For this reason, they were all included in the control group. By contrast, 56·0 % of women (*n* 302) were HPV-positive and further classified as cases (*n* 127) and controls (*n* 175) according to histological diagnosis. Thus, the study population was finally split into 127 cases and 412 controls, independent of their HPV status. The characteristics of women according to HPV status and histological diagnosis have already been described elsewhere^(12,25,34)^. However – to better understand the current analysis – it is worth underlying that cases were younger (*P* < 0·001) and heavier (*P* = 0·012) than controls. Moreover, they were also more likely to be smokers (*P* = 0·001), nulliparous (*P* = 0·011) and users of oral contraceptives (*P* = 0·039).

### Intake of foods and nutrients with antioxidant and anti-inflammatory potential

**Fig. 2:**
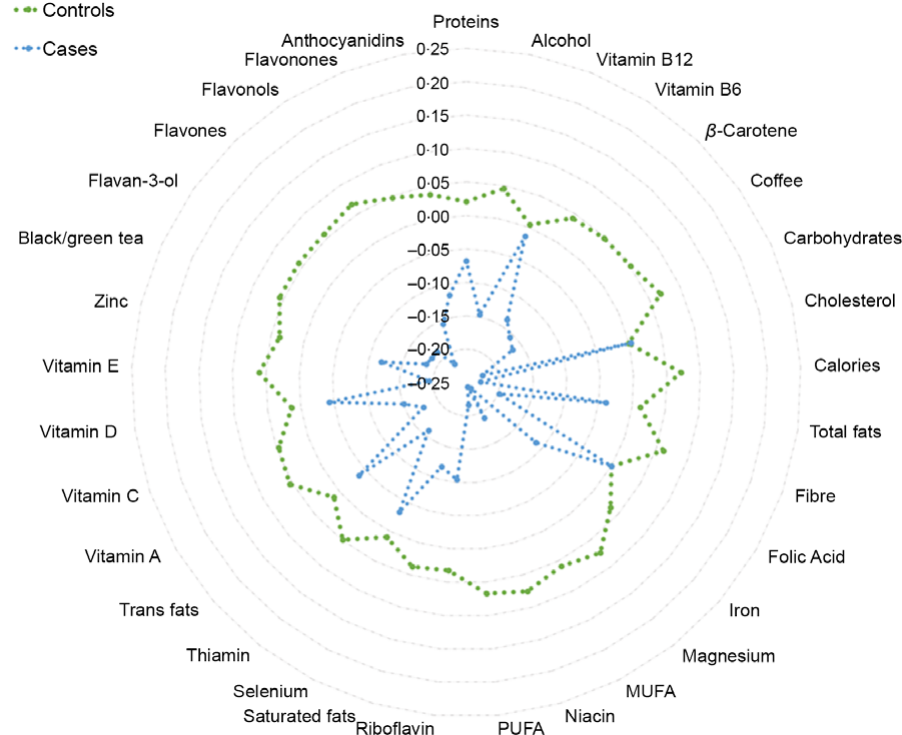
Radar plot illustrating dietary intakes between cases and controls. This plot shows the *Z*-scores of dietary factors and their comparison between cases (CIN2 + women; blue line) and controls (women with diagnosis of normal cervical epithelium or CIN1; green line). CIN, cervical intraepithelial neoplasia

We first observed that cases reported lower intakes of energies and other foods or nutrients with antioxidant and anti-inflammatory potential (i.e. coffee, black and green tea, carbohydrates, fibre, PUFA, vitamins A, B_6_, C, and E, *β*-carotene, niacin, Mg, and flavonoids; Fig. 2 and Table 1). However, none of the above-mentioned foods and nutrients was significantly associated with histological diagnosis after adjusting for multiple comparison.

**Table 1:** Comparison of daily dietary intakes between controls and cases

Foods and nutrients	Controls		CIN2+		*P*-value
Proteins (g)	71·0	64·9	65·5	50·6	0·381
Alcohol (mg)	3·2	5·1	2·2	4·8	0·059
Vitamin B_12_ (μg)	8·6	10·1	8·4	8·0	0·851
Vitamin B_6_ (mg)	1·9	0·7	1·8	0·6	0·030
*β*-Carotene (mg)	1·7	1·9	1·3	1·3	0·044
Coffee (g)	10·2	6·0	8·9	5·9	0·033
Carbohydrates (g)	304·9	288·1	225·4	198·0	0·004
Cholesterol (g)	0·2	0·2	0·2	0·1	0·972
Calories (kcal)	2016	546	1859	442	0·003
Total fats (g)	64·1	42·0	62·0	36·0	0·610
Fibre (g)	10·2	13·2	7·0	8·4	0·010
Folic acid (μg)	188·5	111·7	188·5	115·3	0·999
Fe (mg)	13·4	5·0	12·7	4·7	0·146
Mg (mg)	297·5	84·2	271·9	68·1	0·002
MUFA (g)	49·6	17·3	45·3	16·4	0·014
Niacin (mg)	41·5	20·3	35·1	18·3	0·002
PUFA (g)	14·6	5·8	13·0	4·8	0·005
Riboflavin (mg)	4·7	3·7	4·2	2·8	0·180
Saturated fats (g)	3·7	1·4	3·5	1·4	0·125
Se (μg)	59·9	51·6	57·9	42·0	0·680
Thiamin (mg)	1·5	0·5	1·4	0·4	0·076
Trans fats (g)	1·2	1·5	1·1	1·3	0·625
Vitamin A (mg)	1084·5	553·1	961·7	454·2	0·023
Vitamin C (mg)	122·5	90·7	105·6	7·6	0·049
Vitamin D (μg)	4·8	3·5	4·5	5·3	0·581
Vitamin E (mg)	6·7	5·4	5·4	3·7	0·012
Zn (mg)	9·0	2·8	8·6	3·0	0·124
Black/green tea (g)	6·3	12·6	3·4	7·8	0·017
Flavan-3-ol (mg)	75·3	144·4	42·5	89·8	0·016
Flavones (mg)	1·0	1·1	0·7	0·9	0·016
Flavonols (mg)	34·5	52·7	20·6	33·8	0·005
Flavonones (mg)	36·5	73·9	22·1	56·9	0·045
Anthocyanidins (mg)	54·3	118·3	36·8	99·4	0·132

Daily dietary intakes are reported as median (interquartile range) and compared using the Mann–Whitney *U* test between women with CIN2 or more severe lesions (CIN2+) and those with diagnosis of CIN1 or normal cervical epithelium (controls).

### Composite Dietary Antioxidant Index

Next, we investigated the synergistic effect of dietary antioxidants using the CDAI, on the basis of which women were classified into tertiles. We found that women with higher CDAI were older (*P* < 0·001) and heavier (*P* < 0·001), less educated (*P* = 0·001), more likely to have children (*P* < 0·001), and less prone to use oral contraceptives than those with lower dietary antioxidant intake (1st tertile). We also noted a lower proportion of HPV-positive women among those with higher CDAI (*P* < 0·001) (Table 2).

**Table 2:** Comparison of population’s characteristics across tertiles of Dietary Inflammatory Index (DII) and Composite Dietary antioxidant Index (CDAI)

Characteristics	DII							CDAI						
	1st tertile		2nd tertile		3rd tertile		*P*-value	1st tertile		2nd tertile		3rd tertile		*P*-value
Age, years	51·0	7	40·0	5	29·0	6	<0·001	30·0	8·0	40·0	9·0	50·0	10·0	<0·001
Smokers	31·1 %		40·2 %		51·1 %		0·001	46·6 %		41·7 %		34·1 %		0·052
BMI, kg/m^2^	23·5	4·8	22·8	4·9	20·8	4·3	<0·001	21·1	4·1	23·0	5·0	23·4	5·2	<0·001
BMI categories														
Underweight	1·1 %		7·3 %		15·7 %		<0·001	14·0 %		7·3 %		2·8 %		<0·001
Normal weight	58·1 %		64·6 %		69·7 %			70·9 %		61·8 %		59·6 %		
Overweight	30·2 %		18·5 %		9·6 %			11·2 %		20·8 %		26·4 %		
Obese	10·6 %		9·6 %		5·1 %			3·9 %		10·1 %		11·2 %		
Employed	46·7 %		45·8 %		46·9 %		0·976	46·4 %		49·4 %		43·6 %		0·537
Having children	92·8 %		78·8 %		35·2 %		<0·001	42·5 %		72·8 %		91·6 %		<0·001
Low educational level	50·0 %		40·8 %		25·8 %		<0·001	28·5 %		40·6 %		47·5 %		0·001
Oral contraceptive use	2·8 %		10·1 %		15·6 %		<0·001	12·8 %		11·7 %		3·9 %		0·007
Supplement use	15·6 %		14·5 %		6·7 %		0·020	9·5 %		11·7 %		15·6 %		0·199
High-risk HPV infection	38·3 %		59·2 %		70·9 %		<0·001	69·8 %		56·7 %		41·9 %		<0·001

Data are reported as median (interquartile range) or percentage (%) and compared using the Kruskal–Wallis or the chi-square tests.

### Dietary Inflammatory Index

Similarly, we computed the DII and categorised women according to its tertile distribution. In this case, women with higher DII were younger (*P* < 0·001) and thinner (*P* < 0·001), less likely to have children (*P* < 0·001), more likely to be smokers (*P* < 0·001), more educated (*P* < 0·001), more prone to use oral contraceptives (*P* < 0·001), and less prone to use supplements (*P* = 0·020) than those with lower DII. With respect to HPV status, we found more HPV-positive women among those with higher DII (*P* < 0·001) (Table 2).

### The associations of dietary antioxidant and inflammatory indexes with cervical cancer

**Fig. 3:**
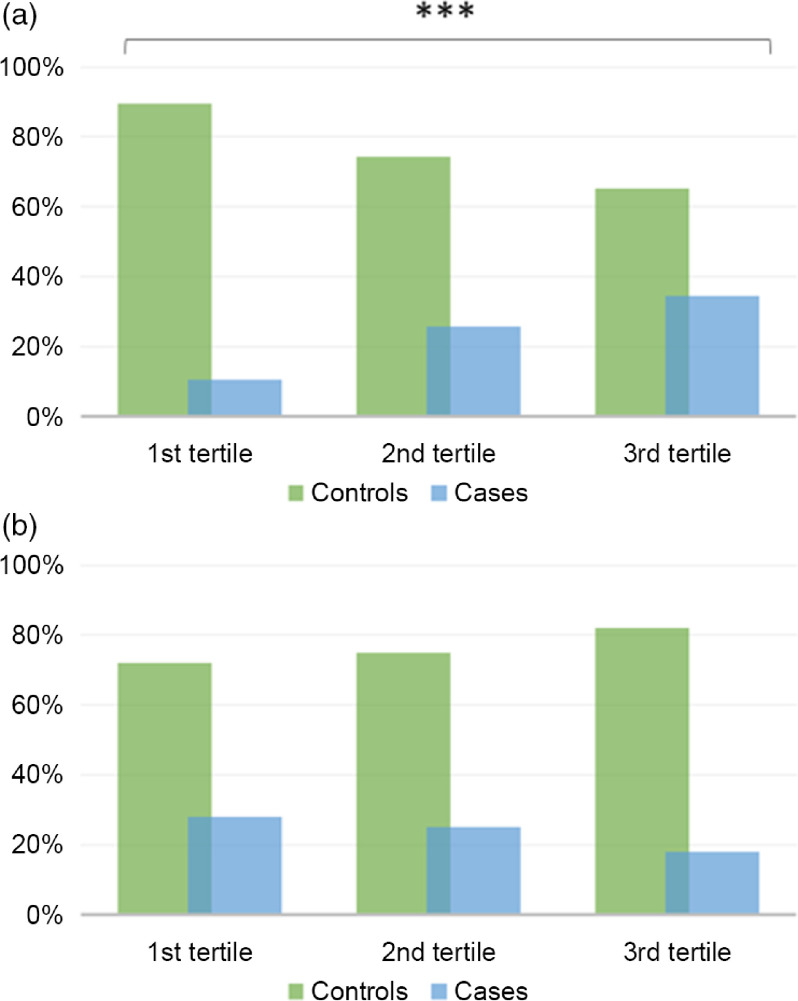
Proportions of cases and controls across tertiles of Dietary Inflammatory Index (a) and Composite Dietary Antioxidant Index (b). The bars represent the proportion women with CIN2 or more severe lesions (cases; blue bars) and those with diagnosis of normal cervical epithelium or CIN1 (controls; green bars). ***P-value<0·001 based on the chi-square test

In line with findings presented above, we next sough to evaluate the associations of CDAI and DII with the diagnosis of CIN2, CIN3 or carcinoma *in situ*. As depicted in Fig. 3(a), the proportion of cases decreased from the 1st tertile to the 3rd tertile of CDAI. However, this difference was not statistically significant (*P* = 0·070). In fact, also considering the effect of covariates, we failed in demonstrating an association between CDAI and the diagnosis of CIN2 or more severe lesions (Table 3). In contrast, the proportion of cases significantly increased from the 1st tertile to the 3rd tertile of DII (*P* < 0·001) (Fig. 3(b)). Accordingly, after adjusting for age and HPV status, women with medium or high DII (2nd or 3rd tertile) had higher odds to be diagnosed with CIN2 or more severe lesions than those with low DII (1st tertile) (OR = 2·02; 95 % CI 1·05, 3·88; *P* = 0·035 and OR = 2·51; 95 % CI 1·33, 4·73; *P* = 0·004, respectively). Notably, these relationships remained significant further adjusting for age, HPV status, educational level, BMI, smoking status, parity, use of oral contraceptives and supplements (OR = 2·15; 95 % CI 1·11, 4·17; *P* = 0·024 and OR = 3·14; 95 % CI 1·50, 6·56; *P* = 0·002, respectively) (Table 3). No interactions of DII or CDAI with other covariates were evident (*P*-values >0·05).

**Table 3:** Association of Dietary Inflammatory Index and Composite Dietary antioxidant Index with CIN2 or more severe lesions

Regression models	Dietary index group	OR	95 % CI	*P*-value
Dietary Inflammatory Index				
Model 1	1st tertile	Ref		
	2nd tertile	2·02	1·05, 3·88	0·035
	3rd tertile	2·51	1·33, 4·73	0·004
Model 2	1st tertile	Ref		
	2nd tertile	2·15	1·11, 4·17	0·024
	3rd tertile	3·14	1·50, 6·56	0·002
Composite Dietary Antioxidant Index				
Model 1	3rd tertile	Ref		
	2nd tertile	1·18	0·70, 2·01	0·532
	1st tertile	1·12	0·62, 2·00	0·711
Model 2	3rd tertile	Ref		
	2nd tertile	1·20	0·69, 2·09	0·523
	1st tertile	1·17	0·61, 2·23	0·637

Results of the logistic regressions are reported as OR and 95 % CI.

Model 1 was adjusted for age and HPV status.

Model 2 was further adjusted for educational level, BMI, smoking status, parity, use of oral contraceptives and supplements. Reference groups (Ref) were the 1st tertile for the Dietary Inflammatory Index and the 3rd tertile for the Composite Dietary Antioxidant Index, respectively.

## Discussion

To the best of our knowledge, the current cross-sectional study is the first evaluating the prevalence of CIN2 or more severe lesions associated with DII and CDAI in a Mediterranean population. These are respectively two of the most used indexes for dietary inflammatory potential and cumulative antioxidant intake in epidemiological research. For this purpose, we first evaluated dietary intakes of foods and nutrients with antioxidant, anti- or pro-inflammatory potential among women with abnormal Papanicolaou test results. In this population, we previously demonstrated that the adherence to a Mediterranean-like dietary pattern – characterised by high intake of legumes, vegetables and olive oil – was associated with lower risk of CIN2 or more severe lesions^(12)^. Here, we added to this evidence, showing that women with CIN2 or more severe lesions had a lower intake of energies and of other dietary factors with antioxidant and anti-inflammatory potential (i.e. coffee, black and green tea, carbohydrates, fibre, PUFA, vitamins A, B_6_, C, and E, *β*-carotene, niacin, Mg, and flavonoids) than their counterpart with negative histological diagnosis or CIN1. In line with these findings, we hypothesised that the cumulative antioxidant intake and/or the inflammatory potential of diet might be associated with the odds of having CIN2+ lesions.

With respect to the CDAI, we reported that the proportion of HPV-positive women decreased with increasing antioxidant score, both here and in a previous study on women with normal cervical cytology^(25)^. In fact, dietary antioxidant intake might counteract oxidative stress and DNA damage induced by HPV persistence^(9,43)^, producing a cellular environment that could help viral clearance^(11,44)^. Yet, despite it has been suggested that antioxidants also prevents CC progression^(44)^, we did not find an association between CDAI and CIN2+ status after adjusting for HPV status and other covariates. This should encourage further research to understand if antioxidants exert their protective role in the entire carcinogenesis process or, on the contrary, if they intervene only in the first phase^(25)^.

In the current study, we also evaluated the DII, a cumulative score based on the anti- and pro-inflammatory potential of foods and nutrients^(42)^. We first noted that the proportion of HPV-positive women increased with increasing dietary inflammatory potential. But even more interestingly, we demonstrated that women with higher DII – and hence women with a pro-inflammatory diet – were more likely to have CIN2 or more severe lesions than those with lower index. The association between DII and CIN2+ status remained significant after adjusting for HPV status and other covariates. This was consistent with the study by Sreeja and colleagues^(26)^, which represented the first attempt in this field of research. Our findings, together with those Sreeja and colleagues^(26)^, indicated that dietary inflammatory potential might modulate the risk of CC independent of HPV infection and other risk factors (e.g. age, cigarette smoking, parity and use of oral contraceptives). Looking at other types of cancer, it has already been shown that higher DII was associated with the risk of other cancers, albeit with some variations related to study design and type of cancer^(45–47)^. For instance, Shivappa and colleagues concluded that a pro-inflammatory diet was associated with increased risk of bladder cancer^(46)^. By contrast, Zheng and colleagues did not support a direct association of DII with pancreatic cancer; however, their results differed according to different follow-up times^(45)^. In 2017, the meta-analysis by Fowler and colleagues tried to solve some controversies. Interestingly, upon stratification by cancer type, they demonstrate a significant association with breast, colorectal and lung cancers^(47)^.

Although no other study has ever considered the relationship with CC, our results are in line with the notion that healthy foods, nutrients and dietary patterns might reduce the risk of CC. By contrast, the consumption of high-energy, high-fat and processed foods might increase the risk^(12)^. From a biological point of view, a previous cross-sectional study demonstrated that a pro-inflammatory diet increased serum levels of inflammatory biomarkers (e.g. tumour necrosis factor-*α*, and interferon *γ*, IL-1, and IL-2) among healthy people^(48)^. Indeed, impaired cell-mediated immune response via deregulation of immune system mediators might promote CC progression. However, other physiological and molecular mechanisms could be involved, motivating at least partially the protective effect of foods and nutrients against CC. For instance, it has been shown that foods and nutrients might interact with the DNA methylation process^(36–38,49–51)^, resulting in aberrant DNA methylation of retrotransposable elements associated with the risk of CC (e.g. long interspersed nuclear elements 1; LINE-1)^(27,34,52)^. However, this field of research is continuously expanding, with several factors and diseases associated with LINE-1 methylation^(53–57)^. Thus, further research is needed to understand molecular mechanisms underpinning the protective or detrimental effect of dietary factors against CC also considering genetic susceptibility and epigenetic markers^(6,58)^.

The current study had some limitations to be considered, such as its cross-sectional design that hindered to evaluate the causality of observed associations. Moreover, the inclusion criteria – and therefore the selection of women who were screened for HPV and tested through colposcopy – cannot exclude potential selection bias. However, this was a convenience sample that allowed us to test the association with high-grade CIN and to adjust our findings for HPV status. With respect to dietary assessment, we used a FFQ that might be affected by measurement errors and inaccuracies. To partially manage reporting bias, we excluded from the analysis all women with the lowest and highest values of total energy intake (i.e. those in the 5th and 95th percentiles). We also adjusted food and nutrient intakes for total energy intake prior to further analyses. Although the FFQ used in this study allowed us to obtain the intake of thirty-three of the forty-five dietary factors included in the DII, the residual effect of other foods and nutrients with anti- or pro-inflammatory potential was not evaluated. Moreover, the DII was characterised by intrinsic limitations common to all a priori dietary obtained through structured questionnaires (e.g. dependence on memory, incomplete data collection and different thresholds for classification), which also made difficult the comparison with previous studies on other types of cancer. Another weakness of our study was the absence of any sort of validation using biomarkers, which for its part would have allowed to overcome some limits of dietary indexes. It is worth mentioning that the FFQ used in our study collects dietary data related to 1-month prior the recruitment and that it does not take into account any changes related to food cooking. In general, FFQ are relatively simple, cost-effective and time-efficient tools to collect dietary data in large epidemiological studies. However, doubts on their accuracy were raised in the last decades, an issue that is still debated among nutritional epidemiologists^(59)^. Finally, we cannot rule out the possible impact of unmeasured factors, which might be associated with dietary habits or CC risk. However, it is worth noting that our analyses were adjusted for age, HPV status, educational level, BMI, smoking status, parity, use of oral contraceptives and supplements. Thus, further prospective studies evaluating additional factors, as well as inflammatory markers in the serum of patients, should be recommended.

In conclusion, our study provided a comprehensive analysis of foods and nutrients with antioxidant and inflammatory potential among women at risk for CC. While the proportion of HPV-positive women was lower among those with high CDAI, the dietary antioxidant intake was not associated with the prevalence of CIN2 or more severe lesions. In contrast, we demonstrated that women who adhered to a pro-inflammatory diet – specifically those with higher DII – exhibited increased odds of CIN2 or more severe lesions. Although these findings should be confirmed by further research, at the present time they suggested how some foods and nutrients might modulate the risk of CC.
